# Observations on Hydrophobia, with Cases, in One of Which Chloroform Was Administered with a Favorable Result

**Published:** 1849-06

**Authors:** 


### ECLECTIC DEPARTMENT,

### AND SPIRIT OF THE MEDICAL PERIODICAL PRESS.

---

*Observations on Hydrophobia, with cases, in one of which chloroform was administered with a favourable result.* By Samuel Jackson, M. D.' Professor of the Institutes of Medicine in the University of Pennsylvania.—(Read before the College of Physicians of Philadelphia.)

The number of cases of Rabies Canina, or Hydrophobia as usually named, that have occurred within the last year, have invested the disease with an increased interest and importance to the profession. From its unusual prevalence in the canine race, over a wide extent of country, it has presented, to a certain degree, the character of an epidemic in that class of animals. Accidents from the bites of rabid animals have been proportionally numerous, producing in the public, excitement, alarm, and a sense of insecurity from the domestication of the dog, whose almost human instincts have made him the companion, protector, and friend of man, one that never deserts him in the hour of peril, or abandons him in the time of need.

It must be acknowledged that we are much in the dark as respects the pathology and therapeutics of this most formidable disease. Our knowledge of its nature, seat, and mode of treatment, is not more settled than it was when first placed on record two thousand years ago. The uniformly fatal result that has, through all time, attended every perfectly authenticated case of communicated rabies canina, renders its curableness a matter to be questioned. Cases of reported cures are on record, but they all have some defect attaching to them, that vitiates their character. Dioscorides asserted that no one was ever cured of the disease, "Neminem sanatum fuisse ;" and the same opinion has been reasserted by Liston, Moreau, Dessault, Peryrilhe, Le Roux, Blair, Hamilton, and others, who have seen and treated cases of this affection.

Our defective knowledge of this disease is, in a great measure, to be attributed to the limited opportunities for making it the subject of study and investigation. Nevertheless, practitioners, in all periods, have passed a lifetime, without having met with a case of it ; and of those who have encountered it, few have seen more than from one to three, or four cases at most, and those at long intervals. For the satisfactory understanding and correct explanation of any class of phenomena, a continued series of observation, experience, and experiment is indispensable ; and this applies in a special manner to a disease at once complicated, obscure, and intractable, such as rabies canina, or communicated hydrophobia.

From the foregoing considerations, it has occurred to me, that the following cases of this formidable disease that have come under my observation, and were carefully recorded at the time, would not be thought obtrusive, or without some interest to the college.

The subject of the first case was a young man who kept a grocery store in Beach or Schuylkill Front Street, in the year 1834. A month or two previously to the attack, he observed in the middle of the day, a small dog to run into the store, and conceal itself between two barrels. In the evening when about to close his shop, he recollected the circumstance, and intending to remove the animal, he thrust his hand between the barrels, seized it by the nape of the neck, and threw it into the street. In doing this, he received a slight bite on the hand. Nothing more was known of the dog, and there is no positive knowledge that he was rabid. So trifling was the wound, and so slight the impression made on the mind of the patient by the occurrence, that when first interrogated as to his having been bitten, when the symptoms excited some suspicions as to the character of the disease, he denied having suffered such a accident. No scar could be seen.

The following history of the case is extracted from my diary, in which I make a daily record of the cases that I attend. The dissection was made by Drs. Horner, Goddard, and myself, and the record of it is in Dr. Goddard's writing, taken down at the time.

Case of Hydrophobia.—*Monday, March 17th,* 1834.—David Lithgow, aged 30 years, sanguine temperament, light eyes and hair, fair complexion, large frame, well developed chest and limbs ; last Monday exerted himself violently jumping ; got heated ; perspired freely, as he usually does ; felt sore after his exertions, particularly around the upper part of the abdomen ; this painful sensation continued some days. On Saturday assisted in loading his canal boat, but did not exert himself more than in common ; felt on Saturday uneasiness in the back of the neck and shoulders, with numbness of his arms, and great pain. Dr. Beattie, was called in, saw him on Sunday about 10 A. M. The patient was in bed covered up with bed-clothes ; the face then looked flushed, and the neck and chest of a red colour ; but when the clothes were removed, the colour diminished. He was bled to ℥xx. Calomel and opium were given. While the Dr. was bleeding him, he drank water without difficulty.

Soon after the Dr. left him, in attempting to swallow the medicine directed, he could not succeed from spasms in the throat. All attempts after this to swallow fluids or solids, increased the same effects. Dr. B. saw him again in the evening, when he exhibited violent convulsive spasms of muscles of respiration, whenever he attempted to swallow. Gave him sulph. morph. gr. iv, laudanum ℥ss, in an injection thrown high up into the bowels. Dr. B. requested me to see him to-day, (Monday, 17th.) Attended at 11 A. M. The patient was in bed, the perspiration flowing from his face ; pupils contracted ; no unnatural heat of surface ; tongue moist ; very slightly furred ; fauces of natural aspect ; pulse 68, soft and round ; bowels being opened frequently ; eructates flatus ; no nausea ; no pain or uneasiness ; has no disposition to spasms, unless excited by some cause. He attempted to swallow a piece of bread for me, but was almost suffocated in the effort. It was expelled from his mouth with violence. Water sucked up through a stomach tube caused violent spasms. The body was jerked upwards ; he rose on his knees in bed, his face greatly distorted by the spasm. Opening the door, though he does not see it, causes him to cry out. The slightest motion of the air made by waving the hand, or a fan, causes convulsive sobs, as though cold water was sprinkled on his face. No other muscles but those of respiration are affected. Applied six moxas to the back of the neck, and injected far into the bowels gr. iij of sulph. morphia. 5 P. M. Symptoms more moderate ; is heavy, inclined to doze ; cups applied down the spine ; afterwards, blister applied on the spine.

*Tuesday*, 18*th*.—Dr. Beattie remained with the patient all night ; gave him in the night ℥j opium in injection, and early this morning ℈j more. Saw the patient at 9½ A. M. Symptoms greatly aggravated ; was out of bed ; manner agitated ; nervous movement ; profuse perspiration, which has continued from the commencment ; pupils contracted ; pulse smaller and feeble ; skin wet and cold to feel ; tongue moist, and nearly clean ; is constantly hawking and expectorating a visced phlegm ; mind is changed since yesterday ; was then calm, pleasant tempered, collected and cool ; is now irritable, quick in temper, irregular in disposition ; endeavours to control himself, but fails at times to do so ; impatient, very desirous to drink ; says in the intervals of the spasms he feels well, yet often suddenly takes a full, deep inspiration ; appears to suffer, yet when asked to explain those feelings, becomes irritated ; is highly excited by trifles. He was positive he could swallow bread soaked in water , a large piece was given to him. Made many ineffectual efforts to bring it to his mouth.

Face contorted, and whole frame thrown into strong muscular action in the effort, but failed to effect it. He then was given a very small piece. After a violent effort, he succeeded in getting it to his mouth; instantly he was seized with most violent spasms of the diaphragm. He had planted himself on his feet, in the attitude of a boxer, to make the effort. With great difficulty he brought his hand to his mouth, when instantly he was jerked backward several feet, and nearly fell. He was tossed about the room in various directions, until he fell on a low bed, in violent strangling spasms. The paroxysm continued several minutes. The sound he makes, apparently a combination of coughing and hawking, might easily be compared to barking, An enema of camphor ʒj, in an emulsion, was administered. Saw him again at 12½; the spasms had continued to increase in frequency, and augment in force; they now come on without exciting cause. In one of great violence, which had continued some minutes, he had fallen on the bed, insensible, and in four or five minutes after, breathed his last. I entered the room just at the close of the paroxysm; the face was deep blue; the muscles in complete relaxation. He died evidently from asphyxia.

*Autopsy* of David Lithgow, ætat. 30, March 19th, 1834; 22 hours after death.—Body natural, muscles tense, became more so during the examination; joints rigid; skin sallow; face much changed in expression; some cadaverous settling of blood in the back, emphysema of neck, chest and right arm. No evidence of priapism.

*Thorax opened.*—Muscles of a very dark hue, becoming red from exposure to the air. Odour of ether strong and distinct, on cutting the pectoral muscles, before the abdomen was opened; emphysema in anterior mediastinum. *Heart.*—Large coagulum in both ventricles; much of the blood, however, being fluid both in the heart and veins; blood black; not becoming red on exposure; substance of heart as firm as usual; lining membrane and valves healthy; lining membrane of aorta and vena cava healthy. *Lungs.*—Healthy externally; rows of globules of air in superficial veins; those of the lower part being fully distended with it. Parenchyma of lungs emphysematous throughout. Air-cells and bronchia healthy; rather dry.

*Neck opened*.—Palate and velum healthy; fauces and glottis covered with a thick tenacious mucus. A broad belt of a deep purple hue surrounded the pharynx, terminating below, in a well-defined line, on a level with the inferior edge of the cricoid cartilage; above, its termination was irregular, and merged into the colour of the rest of the pharynx. It was about an inch and a quarter wide. *Larynx.*—Lower part of epiglottis scarlet, deeper at the sides; mucous membrane of larynx deep red; mucous membrane of trachea and bronchia same as larynx; small quantity of mucus in trachea thick and puruloid. The trunk of the par vagum was examined, and found healthy.

*Abdomen opened.*—Stomach and intestines distended with gas; stomach contained about half a pint of thick fluid, somewhat resembling black vomit; mucous coat of stomach smooth; not softened. Above the cardiac orifice, patches of ecchymosis and stellated injection; latter in large quantities along greater curvature; a large patch of it about centre of larger curvature, dotted injection interspersed; colour of injection brightened on exposure to the air. *Liver* healthy; gall-bladder filled with bile of natural colour and consistence. *Intestines.*—Mucous coat healthy; contained greenish feces of good consistence. *Bladder* healthy, and distended with urine.

*Head opened.* Adhesion between dura mater and skull cap but slight; capillaries of dura mater injected; vascular trunks filled with air. *Pia mater* congested; large veins filled with air, principally at the base of the brain; arteries of base entirely empty; arachnoid membrane somewhat dry. *Medulla oblongata*, white, firm, and drier than natural; cineritious portion pale; large dots of blood in substance; on cutting it open longitudinally from behind, numerous transverse fissures, filled with blood, were seen. *Pons Varolii* white and firm; numerous red dots; cineritious portion paler than natural, but not so pale as that of the medulla oblongata. Cerebrum appeared healthy. The cineritious portion of the natural hue, and the medullary containing the usual number of red dots. Plexus choroides and vena galeni full of air. Thalami and corpora striata healthy; ʒjss of bloody serum in ventricles. Nervous trunks from base healthy.

Spine not examined.

P. B. GODDARD.

The second case I witnessed, daily, during its progress, but did not attend; it was that of Cornelius Weeks, who died in the Pennsylvania Hospital this fall. A full account of this case was kept by Dr. Sargeant, together with the post-mortem appearances. A statement of them has already been made to the College by Doctor Pepper.

I allude to it now, for the purpose of pointing out the different modes of dying, in this case, and that of Lithgow.

In the case of Cornelius Weeks, the nervous spasms were very violent the first forty-eight hours after his admission, but from the effects of chloroform, internally, as a potion, or from the natural course of the disease, they moderated very considerably. Throughout the duration of the case, the potion, two tablespoonsful of water, or broths were taken, though with strong efforts, at regular intervals. He sank away gradually, in a state of extreme exhaustion, presenting the general aspect and physiognomy of malignant fevers. He died apparently from the direct influence of the rabid virus on the blood and vital forces.

Lithgow, on the contrary, perished suddenly in the midst of a violent spasmodic paroxysm, causing asphyxia.

From the post-mortem appearances, I have always looked on his death as having been the result of the introduction of air into the blood-vessels, interrupting suddenly, the circulation. This may have been accomplished by the spasm forcibly compressing the lungs, distended with air, which could not escape from the firm closure of the glottis. Bichat, in his work "*Sur la vie et la mort,*" relates experiments he performed, that prove, it appears to me, the probability of this occurrence. It was this experiment of Bichat's that, at the time of the dissection, suggested the explanation I have given.

Bichat states that, if a stop-cock be adapted to the trachea of a dog, and a quantity of air somewhat greater than an ordinary inspiration, be pushed into the lungs with a syringe, and an artery be then opened in the leg or foot, there will escape frothy blood from the bubbles of air it contains; and if hydrogen be employed, and a lighted candle be held near, they will inflame, showing that the hydrogen, unchanged, had passed into the circulation.—pp. 354–55.

The third case is that of Mrs. Burrows, which has excited some curiosity and interest, from the recovery of the patient. This very uncommon result is well calculated to cast a doubt on the true nature of the disease, and suggest suspicions whether it was a true case of rabies canina. On this account, I shall lay before the College a somewhat minute and detailed statement of all the circumstances of the case, and leave it with them to decide the question.

Mrs. Burrows is about 30 years of age, rather of full embonpoint, a brunette, with black hair and dark eyes. She has a physically nervous temperament, but possesses a determined character, great resolution, and a flow of spirits that rarely fails. When a girl, she was subject to nervous attacks and spasms. The last severe one, her father and herself inform me, occurred ten years since. She has been married seven years, and has had four children; one she lost last summer; the youngest was nine weeks old, at the period of her attack.

Since her marriage, she has had but one nervous spasm, which took place four years since. It was brought on by the painful attempts to remove a pin she had accidentally swallowed, and which was sticking in the fauces, near the top of the larynx. This was the last nervous spasm she had suffered, prior to the invasion of the disease. Dr. Horner saw her at the time, with her brother, Dr. Cooper, at present, surgeon in the U. S. Army.

In the month of July last, then residing in Cooper Street, Camden, she was at the gate door, with her child, a little girl aged——years. She saw two dogs running up the street; she stepped into the yard alongside the house, leaving the child at the door. She soon after was alarmed by the cries of the child, and the noise of a dog, and running to the door, found one of the dogs had attacked her child. She flew to its rescue, and in saving it, received a bite on the inside of the wrist of the right hand. Two punctured wounds were made by the fangs of the dog, about an inch apart. They were slight, and she did not mention the circumstance to her husband, or pay any attention to them; they healed in a day or two. The dog disappeared, and nothing more has been heard of him.

No inconvenience was experienced from the bites, until the commencement of October, when the slight cicatrices made, became red, slightly tumefied, and painful. In some days after, one festered, which she opened; it discharged a few drops of greenish matter, healed, and gave no further trouble. The other remained hard and painful, and pains extended from it, up the arm, to the shoulder. In a few days, the whole arm became painful and swollen, a small tumour formed on the inner side of the arm, about two inches below the axilla. It did not gather.

During this period, as she occasionally complained of her arm, her friends would inquire of her what ailed it; to which she frequently replied jokingly, that she had heard of a milk-leg, and she supposed hers must be a milk-arm. This is mentioned, to show that her mind was not occupied with the idea of the bites.

On Friday, 27th October, Mrs. Burrows, after coming down stairs in the morning, drank a glass of cold water, as is her custom. She was surprised by a sudden shuddering sensation, but as it passed off, she thought no more of it. In the course of the day, she crossed over to the city to visit her parents. When on the river, particularly on her return, she felt a singular dread and uneasiness at the sight of the water, she could not understand. It left her when she landed. In the evening, feeling unwell, she resolved to bathe her feet before going to bed. When the water was brought, she attempted to try with her hand its temperature. She was instantly seized with a violent shuddering, and sense of dread. Her husband, who was present, laughed at her, and asked whether she had not been bitten by a mad dog. She was fearful of giving him uneasiness, and did not mention the bite she had received in July. Soon after, in attempting to take a drink of water, she was seized with violent spasms of the throat, and a sense of suffocation, to an alarming degree. Dr. Fisher, of Camden, who was her physician, was sent for, and remained with her the greater part of the night, as the spasms continued to recur at intervals. She was treated with acetat. morph. gr. ¼, every two hours. Dr. Cooper, of Camden, also saw her. I received a message, requesting my attendance, and visited her at 2 P. M. While in the parlor down stairs, I heard a peculiar sound that bore some resemblance to a dog's bark. It was remarked that the patient was then in a spasm, as in them she made that noise. When I reached the chamber, the spasm had ceased. Mrs. B. was in bed, in full possession of her senses, conversed with me without hesitation or difficulty, in a pleasant manner. She had no fever; pulse 68 to 70; skin cool; she complained of fullness of the head, which she attributed to the pills of acetat. morph. The adnata were slightly injected; she complained of pain in the neck, and in the throat; fauces appeared dry, and voice hoarse. Right arm swollen, and exceedingly sensitive; epigastrium sensitive to pressure, but it did not cause spasms, or disturb her breathing. Fanning, or waving the hand did not produce spasms, or unpleasant effects. Water poured from a vessel, though unseen, and no previous intimation given of the intention, Dr. Fisher informed me, had caused paroxysms in the course of the morning. I requested her to drink some water, with which she complied immediately. She took a mouthful, but in trying to swallow, a frightful spasm was induced, limited, as it appeared to me, to the larynx and fauces; she appeared suffocating. The diaphragm and abdominal muscles did not participate in it, as I kept my hand on the abdomen to ascertain the fact. She appeared to me, for a short time, to be unconscious, the eyes rolled upwards, but she declared she retained her senses perfectly. It was a violent struggle for breath, but not general convulsion or spasms. There was no salivation or mucus collected in the mouth.

At this visit, I expressed to my colleagues, Drs. Fisher and Cooper, that although the symptoms were somewhat suspicious, yet, taking everything into consideration, I was disposed to look on the affection as a simple nervous one, and probably hysterical.

The following course was agreed on. Sinapism to the epigastrium; cups on back of neck; sinapisms down the spine; enemata of assafœtida ʒiij suspended in water, and chloroform ʒjss in an emulsion, every hour, if required.

*Sunday morning, Oct. 29th.*—Patient apparently better, very cheerful, calm, collected in manner, and gay in conversation. Spasms had continued yesterday, until the evening, when they had ceased, and had not again returned; passed a quiet night, but did not sleep sound. She had drunk water freely, several times. Some difficulty had been experienced in swallowing it, but no spasms were excited. Head more comfortable since the pills were omitted. Throat feels sore, but less painful than yesterday. Skin, pulse and tongue natural. Sensibility entirely lost in the skin of the right arm, below the deltoid muscle; does not feel pinching or touching. She asked me to stick it with my knife, to ascertain whether she could feel that. There is, notwithstanding, deep-seated pain in the course of the nerves. There are also slight spasmodic twitchings of the muscles of the arm. The whole abdomen feels sore and uncomfortable; pressing the epigastrium gave most uncomfortable sensations, and disturbed the respiration, rendering it irregular, but did not cause spasm. In the night, ejected some blood by vomiting; did not coagulate. Bowels have not been opened.

At this visit my first impressions were rather confirmed, than weakened. It was determined to continue the same plan of treatment, and to add the following, with a view of acting on the bowels: ℞.—Mass. hydrarg., ℈j; syr. rhei, ℥j. A teaspoonful every hour. Enema of emulsion of assafœtida, if spasms continued.

The first dose was given at 12 M. The attempt to swallow it brought on violent spasms of larynx and chest, threatening suffocation.

From this time, the spasms occurred with short intermissions, spontaneously, notwithstanding the assafœtida injections; sinapisms to abdomen, and other means resorted to by Drs. Fisher and Cooper.

I saw her at 5 P. M. There was an intermission when I entered the room. She expressed herself as suffering great bodily distress. The right arm was in constant agitation from slight spasms; the right shoulder painful; no sensibility in the forearm. The cicatrix was tumid, red, and sensitive to pressure, though the hand and arm were insensible. She complained of acute pain in both hams. Pressing on the groins, on the calves of the legs, in the armpits, as well as under the knees, excited acute pain. Has sense of distress in throat, chest, heart, and abdomen. Notwithstanding this state of suffering, talks cheerfully, even answered in the same spirit, to some jocose observations, and expressed her full confidence in her attendant's skill. Without her observing it, I placed my hand near the back of the head, some inches from it, and gently waved it. She was, on the instant, seized with shuddering, followed by strangling spasms of the larynx, fauces, and of the chest, arresting respiration, followed immediately by spasms of the trunk, in which she was tossed about the bed, gnashing her teeth, and plunging her head into the pillows, and bed-clothes, biting and tearing them.

Chloroform was sent for. It was obtained from an apothecary in the neighborhood. When procured, as no sponge was at hand, I soaked a rag with it, and seizing her by the back of the neck, attempted to hold it near her mouth. The inhalation was imperfect; as the spasms kept the patient in constant motion, and she was making plunging efforts to seize the rag with her teeth, some caution was required to avoid being bitten.

A sponge was then procured, and the inhalation was more effectually performed; as the effect took place, and the spasms were mitigated, the patient assisted herself to hold the sponge to her mouth. In a few minutes, the full effect was produced, and she fell perfectly insensible, every muscle in perfect relaxation, and the respiration easy and natural. An enema was now administered, consisting of pulv. ipecac. composit. ℈ij; chloroform ʒij, in starch water.

The medical attendants retired to another room, where the excision of the cicatrix was talked over and determined on.

After returning to the room, while sitting by the bed-side, she suddenly addressed me, saying, "Dr. Jackson, what is my disease?"—"Nervous spasms," I answered.—"I know that, but what causes these spasms?"—"Many causes of various nature, may give rise to them."—"That is true, but is not that the cause," putting her left forefinger on the cicatrix on the right wrist, "Is it not that?"—"Most probably it is."—"Why not, then, cut it out? why not, if necessary, take off my arm? I can bear it, I have nerve for anything."—"Cutting it out is precisely what we have concluded, just now, to do, but it so happens, we have no instrument with us."—"Well, take your penknife, I won't flinch."—Dr. Cooper, who had stepped out, returned with a venerable scalpel that had evidently not been in service for a long time, and a tenaculum. I hooked up the cicatrix, and with some effort succeeded in excising the skin surrounding the cicatrix. This rude surgery was borne well. She then said, "Do you not think it would be better to apply caustic to the cut?"—"A good suggestion," I replied, and immediately applied caustic liberally over the whole surface. A poultice of pulv. ulm. rubr. was directed to be applied.

The excision of the cicatrix was hardly completed, when a spasm came on. The chloroform was immediately administered with the sponge, its full effects were induced, and she again became insensible. She was some time in this state; as she was recovering from it, she raised herself slowly on her knees, and with her eyes intently gazing, and her arm stretched upwards, she addressed the vision of her lately lost child. When she had entirely recovered, she related the vision she had seen.

It was agreed that the chloroform should be given as soon as a paroxysm was observed coming on, that in the course of the night, another enema, similar to the last, should be administered, if the spasms continued to recur, and calomel gr. xx. should be given to relieve the bowels.

*Monday, Oct. 30th.*—At my visit this morning, found her better, calm, and cheerful; pulse 96; temperature of skin natural. Tongue moist, slightly coated. I was informed that spasms had continued to recur, from the time I left her until midnight. Many were exceedingly violent. The chloroform had been timidly administered. As the spasms appeared to yield, the chloroform was withdrawn, from an apprehension of some ill consequences from using it so constantly. The patient, as soon as the spasms would permit her to articulate, would call for more, and urge its use. After 12 o'clock, the spasms were subdued so much, that instead of being instantaneous, she had a warning of their approach, when a few inhalations arrested their further development.

Was sick in the night, and vomited more blood, which remained liquid. Throat feels sore, voice is hoarse; abdomen uncomfortable, and slight pressure distressing; cannot bear the weight of the bed-clothes on it. Pressure on the calves, under the knees, groins, and arms, very painful. Bowels have not been moved. In the confusion, from the conflict with the spasms, the calomel directed, had not been given. No feeling in right arm. It is paralyzed, but is often affected with tremulous spasms.

Mrs. B. is naturally near-sighted. Her father assured me she had been so from early youth. She was unable to distinguish the features of a person standing at the foot of the bed. Her sight is now quite acute. The shutters are bowed, and the curtains drawn, as the light is offensive, yet she sees a pin sticking in the paper, on the opposite wall of the chamber, distant at least twelve feet.

The hearing is equally acute, though her hearing is rather dull in health. Yesterday, when the medical attendants were in the parlor beneath the chamber, the stairway opening into a small entry communicating with the bed-room, she heard the conversation below, and repeated parts of it to those with her at the time.

She remarked to me, that her throat felt so uncomfortable and dry, that she wished it could be greased inside, with a feather. I suggested to her to take some oil of butter, to which she assented. It was prepared and brought to her in a silver spoon; but as soon as the glitter of the metal caught her eye, she was taken with a strong shuddering, and spasmodic action of the throat and face. The oil was then placed in a small toy-cup; she received it in the mouth without difficulty, but in attempting to swallow it, a spasm came on. I called to her to spit it out; but she made another effort, when most of it was expelled, and a strong spasm was induced. The chloroform on a sponge, was brought under her mouth, a few inhalations produced partial insensibility and relaxation, and the paroxysm ceased.

Calomel (gr. xvi) was given. Pills are swallowed without difficulty, and crackers can be chewed and swallowed.

6 P. M.—No complete paroxysm since morning; several times spasms were threatened, but arrested immediately by chloroform inhaled. This afternoon, her father seeing a fly about to light on her face, waved his hand to drive it away. This excited a spasm, checked, however, by chloroform. The looking-glass, and other shining objects in the room, were covered over. The glitter distressed her. The windows were also kept down; she could not bear the air to blow upon her.

I inquired of her what had been, and were, her feelings; she said it was difficult to describe them, but they were more like a dread of something, she knew not what, than any other feeling. Her mind is tranquil; she converses cheerfully; being a Catholic, she has observed the religious obligations of her faith, and is fully prepared for any event.

The wound is discharging freely a thin serous fluid. The arm feels, she says, as though sensation was returning in it.

Bowels have not been opened, or urine passed. Directed a purgative enema, and after evacuations, pulv. ipecac. comp. ℨss, in injection. Chloroform *pro re nata*.

*Tuesday* 31*st.*—Had passed a comfortable night; bowels and bladder had both been relieved last evening, and again this morning. Had taken the Dover's powder injection. Twice spasms had been excited in the evening; once by a young girl coming into the room, and approaching the bed with a glass of water in her hand; the other, by an attendant, without thinking of it, bringing a basin of water into the room; each time chloroform arrested the spasms.

The wound discharges freely; supuration has commenced; sensibility has returned to the arm; pressure on the calves, beneath the knees, in the groins, and armpits, much less painful.

She took last evening, some ice-cream, and repeated it this morning; she has taken also some milk this morning. The uneasiness of throat greatly abated; epigastrium less sensitive; bears pressure without the same distress. She informed me this morning, that during the violence of the attacks, a feeling appeared to start from the cicatrix, ascend the arm, pass down the chest, and strike intothe stomach; but that now the feeling appears reversed, and seems to pass from the stomach into the arm, and descends into the wound.

The chloroform is used whenever there are threats of spasms from uneasy sensations. Repeat the enema of Dover's powder.

I enquired of her whether there was any difference between the attacks she had suffered from the last few days, and those I had understood she was formerly subject to. She said there was; they were wholly dissimilar. I asked in what respect. There is a difference, she remarked; in the former attacks I was generally unconscious; I knew no one about me, what was said, or what was doing. When I came to myself, I did not know that anything had happened to me. In these last I had my consciousness entire. I knew every one, heard all that was said, and I knew all that was doing. There is also this difference. In my old attacks, bandages were tied tight around my stomach, and pressure made, which always gave me relief in the milder attacks; in these last, I could not bear the slightest pressure on the stomach; the bed-clothes oppressed me.

*Nov.* 1*st.*—Was restless in the night. The hand, wound, and arm more painful; the edges of the wound pale and unhealthy; discharge thin and sanious. Directed to be dressed with ungt. resinæ flav. Abdomen and epigastrium are no longer sensitive, and seat of uncomfortable sensations; bowels relieved. This morning has taken ice-cream and milk. She has swallowed three or four raw oysters; complains of thirst, and wishes a trial of drinking water. Some was brought to her, and she took a large draught. A slight tremor only was produced, followed by a sense of glow, and suffusion of the face; continue milk, ice-cream, and raw oysters. At night the usual enema of Dover's powder. Chloroform has been discontinued.

4*th.*—Has continued free from spasms; arm been painful. To-day was brought in a carriage from Camden, to her father's residence in Market street above Ninth. Saw her after arrival. She was in sitting-room down stairs, resting herslf. At 9 P. M. I was sent for to see her. In carrying her up stairs to her chamber, she had fainted. She continued from fifteen to twenty minutes in that state. She revived soon after I left the room, when, as usual, she commenced with me a cheerful conversation.

She informed me that she had lost her milk during her illness, and will be compelled to get a nurse.

*5th.*—Has rested well; feels better though feeble; arm less painfal.

*23d.*—Have not seen Mrs. Burrows until this evening, at 10 P. M., when her brother Doctor Cooper, of the U. S. Army, urged me to visit her immediately.

Since last report, her general health has been good. The arm has remained painful; the pain appears to be confined to the ulnar nerve in the forearm, but the whole shoulder is painful. On the 17th, Doctor Fisher saw her, and as she was that day feeling very uncomfortable, with increased pain of the arm, and the wound was nearly healed, he again applied caustic potash. The slough was thrown off to-day. The pain of the arm had been increasing for the last two days, and finally, this evening, strong spasms of the arm came on, recurring in paroxysms every ten or twelve minutes, accompanied with sense of numbness. Severe pain existed also in the nape of the neck, extending down the back to the last dorsal vertebra.

I directed a warm poultice with ten grains of powderd opium to be applied to the wound. A pill containing sulph. morph. gr. $\frac{1}{4}$, was ordered to be given every two hours, and a dozen dry cups to be applied along the spine in the neck and back. One spot opposite the third dorsal vertebra was exceedingly sensitive; when a cup was applied to it the right arm was thrown into violent spasms, the forearm was rigidly flexed, and the hand clenched. It continued in this state until the cup was removed.

*24th.*—The pain and spasms of the arm continued nearly all night. Towards morning became less, snd the patient got some sleep.

No spasms of the arm to-day; the course of the nerve is yet tender; a little below the axilla is very sensitive; wound discharging freely; a liniment of extr. of stramonium, aconite, opium, with cerate of oil, was directed to be rubbed on the arm, and the pill to be continued at intervals of four to six hours.

*25th.*—Rested well all night; arm less sensitive; wound looks healthy; omit the pills; continue the liniment.

From this period Mrs. Burrows continued to improve in health. Her milk returned. The wound cicatrized in the second week in December, the pain ceased in the forearm, but the shoulder and axilla continued sensitive, and occasionally painful, into the commencement of January. To the present time she continues to enjoy the most perfect health.

In the three cases of Lithgow, Weeks, and Mrs. Burrows, the symptoms of hydrophobia were strongly manifested. Weeks' case was undoubted rabies canina. I draw a distinction between them. Hydrophobia is a nervous affection, more frequently a nervous symptom, complicating or accompanying other diseases. It may be regarded as nearly pathognomic of rabies from its almost uniform attendance in a greater or less degree on it, and hence is often confounded with it. Hydrophobia may exist, though rarely, as a simple nervous disease; it most commonly is manifested as an hysterical symptom, when it does not belong to rabies.

Lithgow died from hydrophobic spasms, by which asphyxia was induced. Weeks did not die in spasms, or from proper hydrophobic disorder, but in a state of complete adynamia and ataxia, prduced by rabific poisoning. In most of the fatal cases of reported rabies, death appears to have ensued from asphpxia brought on by spasms of the respiratory apparatus. Doctor Marshall Hall has lately stated that death always in hydrophobia occurs in this way. Dr. Physick appears to have entertained the same opinion, as he recommended tracheotomy to be always performed; and the same advice has been reiterated by European practitioners.

In the case reported by Doctor E. Hartshorne, (*American Journ. Med. Sci.*, Oct. 1848, p. 339,) the hydrophobic spasms ceased before death, and the patient was able to swallow liquids. In that case as in Weeks', death resulted from the deadly influence of some virus infection of the blood, and destroying its vital capabilities.

In Lithgow the hydrophobic spasms were more violent than in Weeks; and in Mrs. Burrows they were equally strong and distressing, until checked by the chloroform inhalation.

Mrs. Burrows' case I regard as one of hydrophobia; was it rabies? That cannot be asserted in the absence of all information respecting the rabidness of the dog by which she was bitten. That it was not hysteria, I feel equally confident. I have had to manage numerous cases of that affection in private and public practice, but never have I met with a case in which so few genuine hysterical symptoms were present, or exhibited any approach to the train of phenomena that were present in Mrs. Burrows. That the hydrophobic symptoms had their origin in the cicatrix of the bite, I cannot doubt. There was a degree of painful irritation there, more than sufficient to account for the whole train of nervous disorder and symptoms she suffered.

I have seen two fatal cases of tetanus produced by irritating causes incomparably less active. One was an apprentice of Mr. William Fry. He had received a slight scratch in his thigh from the jagged end of a board. Doctor Horner and myself examined the body. The wound had been healed some time. We made a most careful search for some foreign body in the skin, and when about to abandon it, accidentally, I perceived a slight hardness in the facia lata, which was to all appearance perfectly healthy. On splitting it open, there was found a small splinter about one-eighth of an inch in length, and a line in breadth. There was no inflammation, the lad had suffered no pain, no local sign of any injury or disturbance was to be detected; yet the injury and the presence of that trifling foreign body, had been sufficient to cause a fatal disease of the nervous system.

In the other case, a small piece of leather from the shoe, had been forced beneath the nail of the great toe, by a projecting nail, against which it had been struck. No inflammation, or swelling, or suppuration of the toe had taken place, but death ensued from tetanic spasms.

The excision of the cicatrix in Mrs. Burrows' case, and the establishment of a free discharge and suppuration, it is most probable contributed, by removing the exciting cause of the spasms, to the recovery of the patient.

The inhalation of the chloroform unquestionably produced the most beneficial effects in arresting the spasms, and preventing their developement. But it cannot be relied upon as a curative agent in true hydrophobia, or rabies canina. It is an important aid by holding the paroxysms in check, and may prevent death from their violence, or their long duration.

In the above three cases, the most striking phemomenon, was the exalted and perverted action of the excito-motory function of the medulla-oblongata (hyperdynamia and ataxia) connected with the respiration and deglutition. In all the well-reported cases of rabies canina and communicated hydrophobia, the same phenomena have prevailed. Impressions and excitement communicated to it through the eye, as shining, or glittering, or strong light; through the ear, as sounds of water, sometimes of wind; from the face, as movement of air over it; from the mouth and fauces, as the touch of water and sometimes of solids, or the efforts to swallow them, throw the respiratory apparatus and the muscles of the throat, into disordered, irregular, convulsive, or spasmodic action. This often is so violent as to produce death from asphyxia.

In all cases of rabies the medulla oblongata, the exciting and governing central nervous organ of the involuntary and automatic muscular actions, is in the above pathalogical state, but varying as to its degree in different individuals, during the first and second stages of the disease. Should the patient survive the two first stages, and pass into the third, a new train of phenomena is then established. With the ataxia of the early periods is combined adynamia, from the breaking down and exhaustion of nerve force; muscular power rapidly fails, and the vital functions depending on muscular action and physical laws, for their maintenance, as respiration and circulation speedily are extinguished.

Before concluding this commuication, already longer than I had intended, there is one remark I wish to make. A case of hydrophobia produces much excitement and interest. The patient is most generally surrounded by curious visitors, professional as well as others. From the excited and perverted state of the nervous functions, the effect is injurious. Spasms are brought on, and agravated by the impressions made on the senses, and by the annoyances he experiences. I was much struck with it in Lithgows' case. Form morning to night his room was crowded by people of the neighbourhood, and professional gentlemen calling to witness a case of so rare an occurrence. Weeks was much distressed and annoyed by the numbers of the gentlemen of the profession that at first crowded into his room, but subsequently this was corrected. He remained alone, or with a single attendant, until the time for administering his remedies, or the period for visiting him by the medical prescribers. I asked him if he had spasms when entirely alone; he told me he had not.

None were permitted to see Mrs. Burrows but members of her family who were her nurses, and her medical attendants. I am convinced that a patient has a better chance of recovery, certainly has much fewer attacks of spasms, and far less suffering, when kept in as quiet a state as possible.

